# The Ohio State Dental Society

**Published:** 1887-11

**Authors:** 


### EDITORIAL.

### The Ohio State Dental Society.

The annual meeting of this body has just been held in Springfield. The meeting was well attended, and the proceedings were both interesting and profitable. Quite a number of papers were read and pretty fully and well discussed. It is encouraging to note the progress that is being made in this part of society work from year to year. There is certainly a growing interest in and appreciation of association work each year. This is true not only of this society, but of all bodies of this kind in the dental profession. And an encouraging feature is that this progress is found in a large degree in the younger members. Our educational facilities account in a measure for this. Of course such results ought to be expected, if our schools are to fulfill their purpose. We hope soon to give a full report of the proceedings.

